# Supplementary figures and images for: A high-quality chromosome-level genome assembly of rohu carp, Labeo rohita, and its utilization in SNP-based exploration of gene flow and sex determination
Source: G3 (Bethesda). 2023 Jan 14;13(3):jkad009. doi: 10.1093/g3journal/jkad009 (PMC9997561; doi:10.1093/g3journal/jkad009)

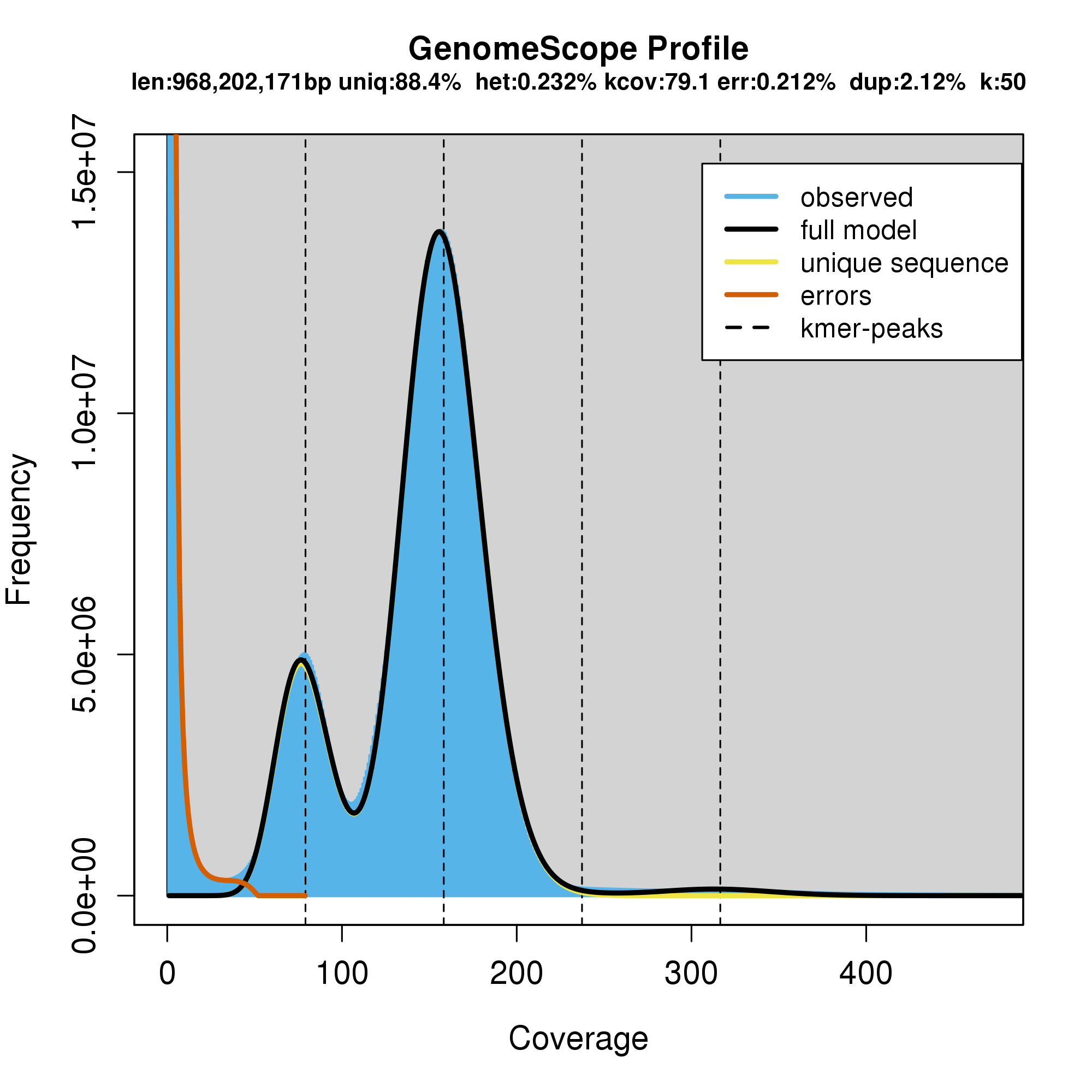

Supplement: jkad009_Supplementary_Data [file jkad009_supplementary_data.zip › jkad009_Supplementary_Data/Figure_S1_G3-2022-403809.png]

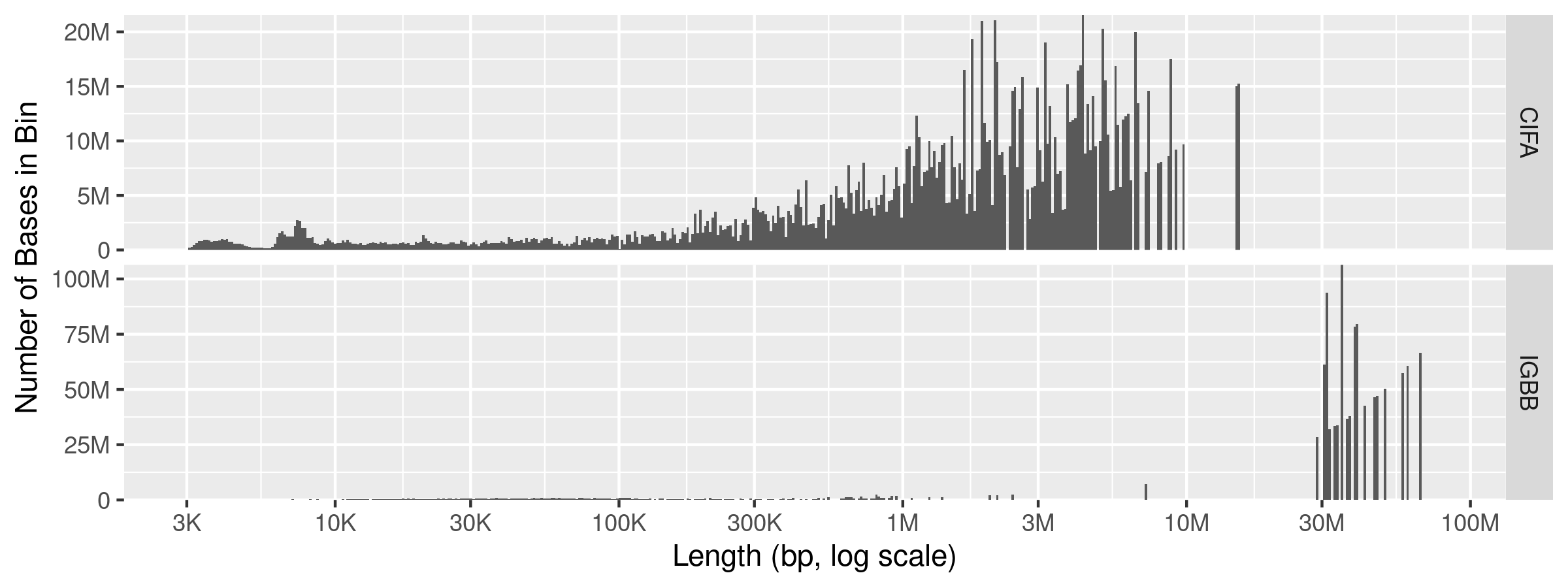

Supplement: jkad009_Supplementary_Data [file jkad009_supplementary_data.zip › jkad009_Supplementary_Data/Figure_S2_G3-2022-403809.png]

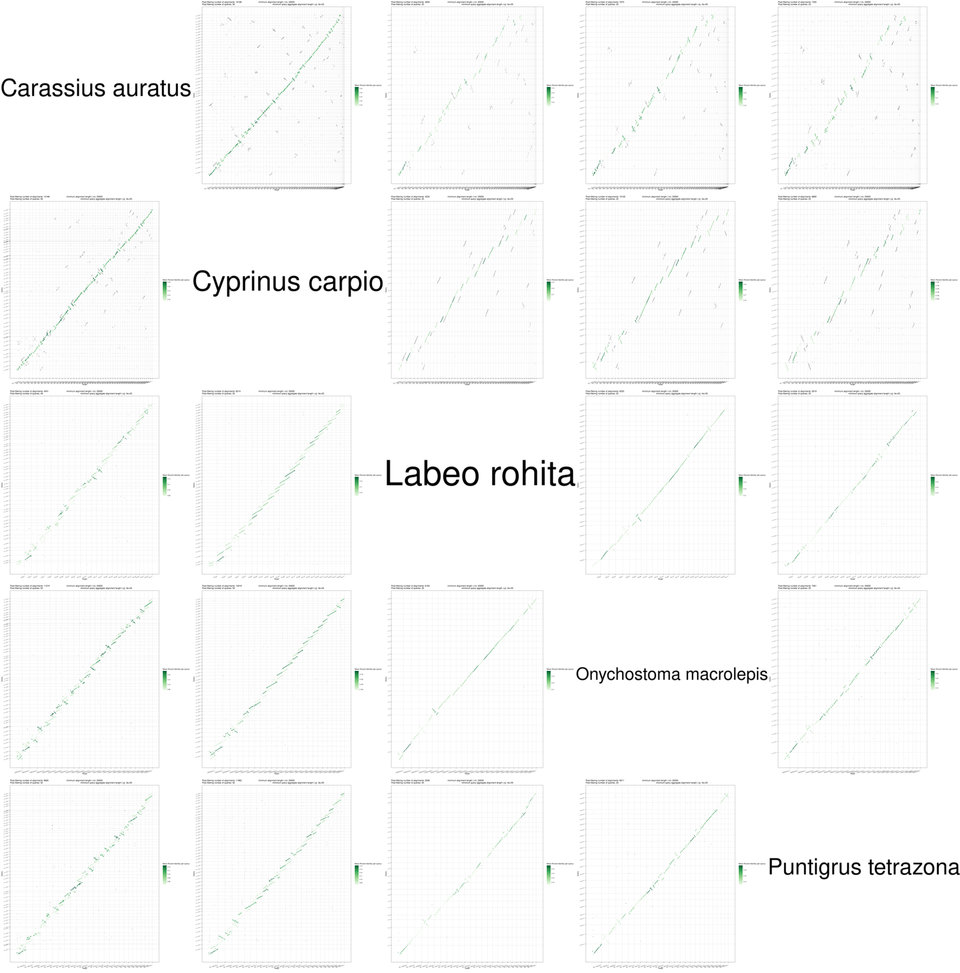

Supplement: jkad009_Supplementary_Data [file jkad009_supplementary_data.zip › jkad009_Supplementary_Data/Figure_S3_G3-2022-403809.png]

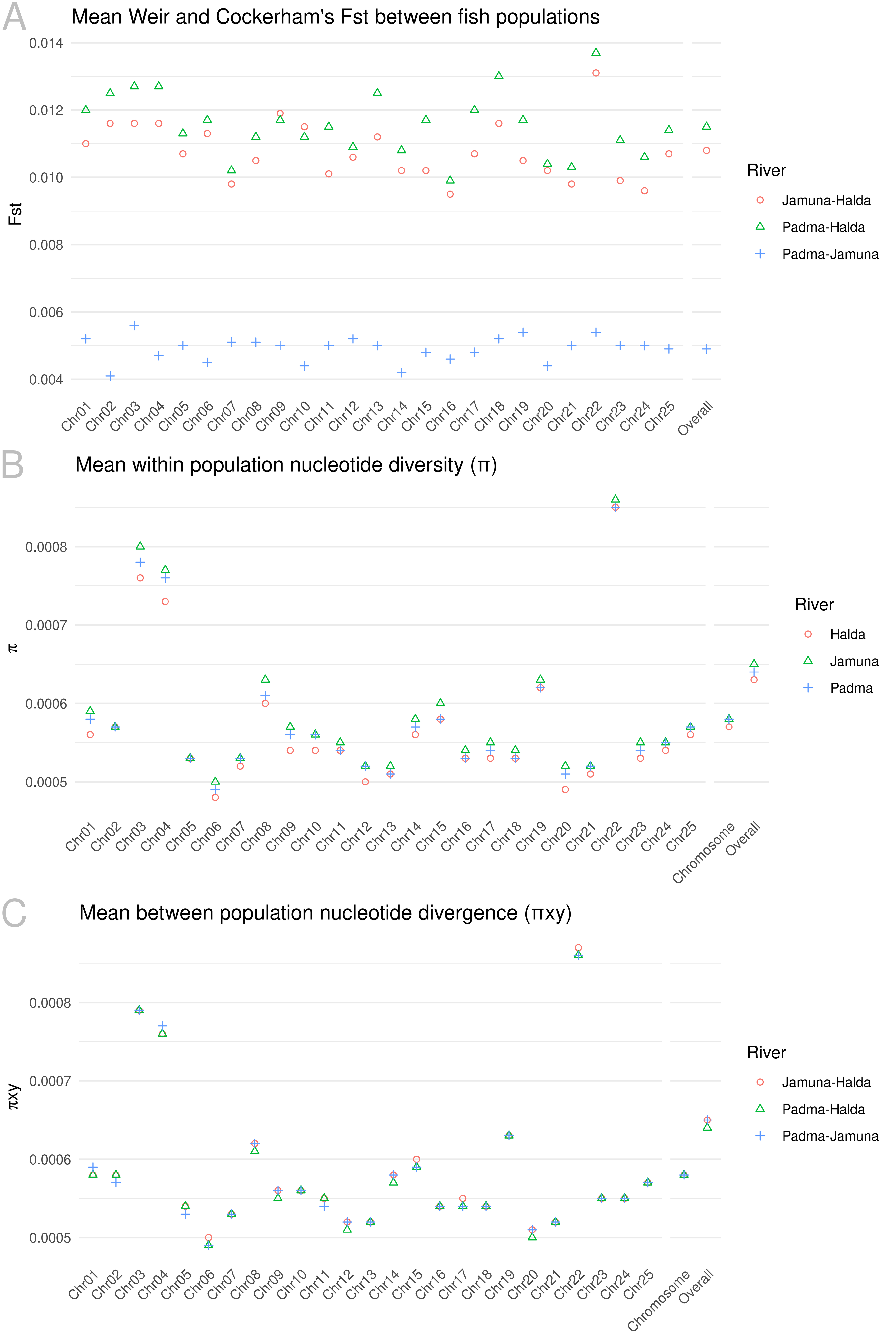

Supplement: jkad009_Supplementary_Data [file jkad009_supplementary_data.zip › jkad009_Supplementary_Data/Figure_S4_G3-2022-403809.png]

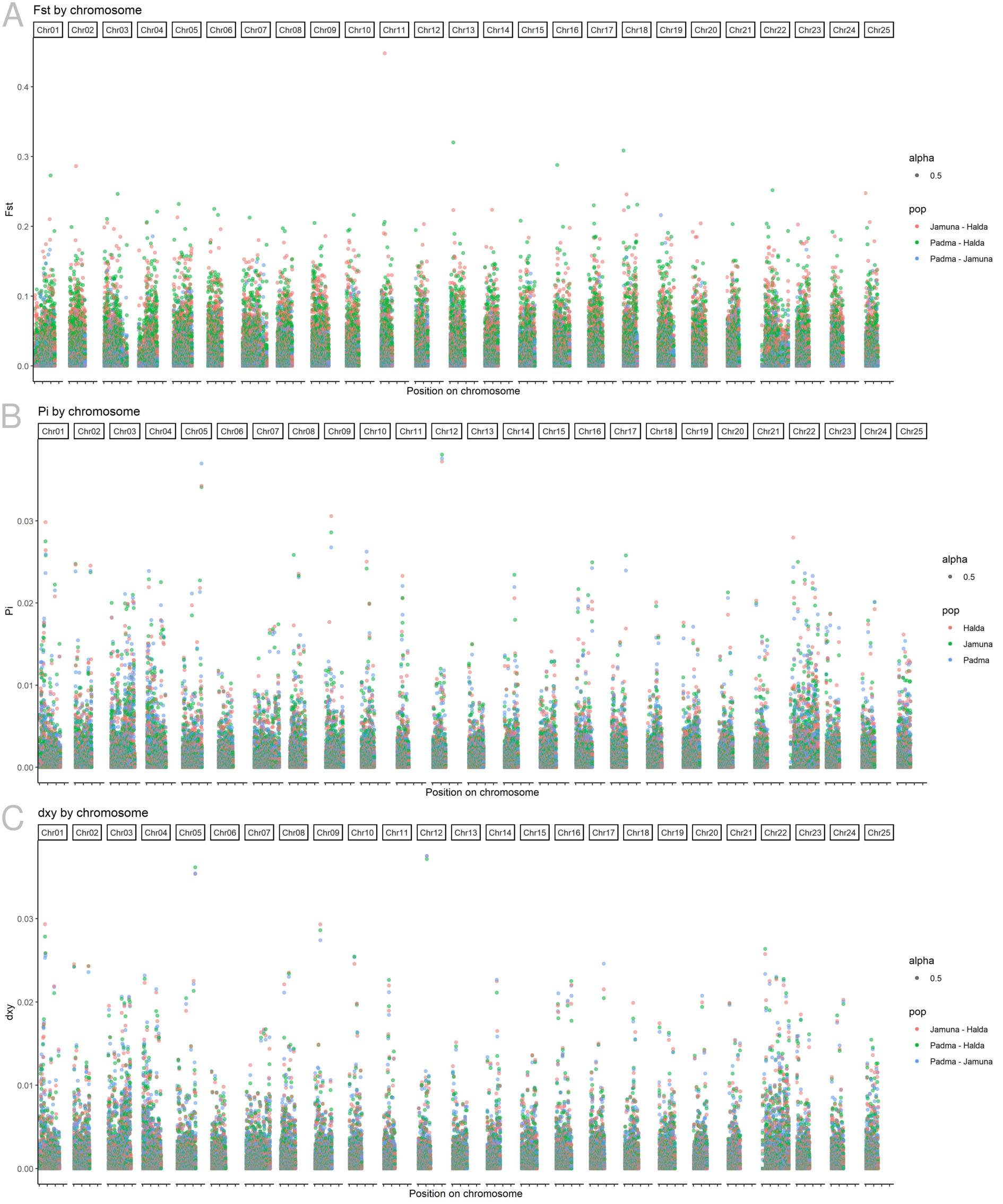

Supplement: jkad009_Supplementary_Data [file jkad009_supplementary_data.zip › jkad009_Supplementary_Data/Figure_S5_G3-2022-403809.png]
